# Mortality and AIDS‐defining events among young people following transition from paediatric to adult HIV care in the UK

**DOI:** 10.1111/hiv.13096

**Published:** 2021-05-03

**Authors:** H Asad, IJ Collins, RL Goodall, S Crichton, T Hill, K Doerholt, C Foster, H Lyall, FA Post, S Welch, A Winston, CA Sabin, A Judd, the Collaborative HIV Paediatric Study (CHIPS) Steering Committee, the UK Collaborative HIV Cohort (UK CHIC) Study Steering Committee, Caroline Sabin, John Saunders, Catherine Mercer, Gwenda Hughes, Sema Mandal, Greta Rait, Samreen Ijaz, Tim Rhodes, Kholoud Porter, William Rosenberg, Alasdair Bamford, Karina Butler, Nigel Klein, Paddy McMaster, Katia Prime, Andrew Riordan, Fiona Shackley, Julia Kenny, Delane Shingadia, Sharon Storey, Gareth Tudor‐Williams, Anna Turkova, Claire Cook, Donna Dobson, Keith Fairbrother, Diana M Gibb, Lynda Harper, Marthe Le Prevost, Nadine Van Looy, Kate Francis, Helen Peters, Claire Thorne, L Thrasyvoulou, K Fidler, J Bernatoniene, F Manyika, G Sharpe, B Subramaniam, R Hague, V Price, J Flynn, A Cardoso, M Abou–Rayyah, N Klein, A Bamford, D Shingadia, S Yeadon, S Segal, S Hawkins, M Dowie, S Bandi, E Percival, M Eisenhut, K Duncan, L Anguvaa, L Wren, T Flood, A Pickering, P McMaster, C Murphy, J Daniels, Y Lees, F Thompson, A Williams, B Williams, S Pope, Dr S Libeschutz, L Cliffe, S Southall, A Freeman, H Freeman, S Christie, A Gordon, D Rosie Hague, L Clarke, L Jones, L Brown, M Greenberg, C Benson, A Riordan, L Ibberson, F Shackley, S Patel, J Hancock, K Prime, M Sharland, S Storey, EGH Lyall, P Seery, G Tudor‐Williams, N Kirkhope, S Raghunanan, Julia Kenny, A Callaghan, A Bridgwood, P McMaster, J Evans, E Blake, A Yannoulias, Jonathan Ainsworth, Sris Allan, Jane Anderson, Abdel Babiker, David Chadwick, Duncan Churchill, Valerie Delpech, David Dunn, Brian Gazzard, Richard Gilson, Mark Gompels, Phillip Hay, Margaret Johnson, Sophie Jose, Stephen Kegg, Clifford Leen, Fabiola Martin, Dushyant Mital, Mark Nelson, Chloe Orkin, Adrian Palfreeman, Andrew Phillips, Deenan Pillay, Frank Post, Jillian Pritchard, Caroline Sabin, Achim Schwenk, Anjum Tariq, Roy Trevelion, Andy Ustianowski, John Walsh, Sophie Jose, Andrew Phillips, Caroline Sabin, David Dunn, Shaadi Shidfar, Chloe Orkin, Janet Lynch, James Hand, Duncan Churchill, Nicky Perry, Stuart Tilbury, Elaney Youssef, Brian Gazzard, Mark Nelson, Sue Wood, David Asboe, Sundhiya Mandalia, Jane Anderson, Sajid Munshi, Frank Post, Ade Adefisan, Chris Taylor, Zachary Gleisner, Fowzia Ibrahim, Lucy Campbell, Abdel Babiker, David Dunn, Shaadi Shidfar, Nicolas da Silva Santamaria, David Chadwick, Kirsty Baillie, Richard Gilson, Nataliya Brima, Ian Williams, Jonathan Ainsworth, Achim Schwenk, Sheila Miller, Chris Wood, Margaret Johnson, Mike Youle, Fiona Lampe, Colette Smith, Rob Tsintas, Clinton Chaloner, Samantha Hutchinson, John Walsh, Nicky Mackie, Jonathan Weber, Farhan Ramzan, Mark Carder, Clifford Leen, Andrew Kerr, Sheila Morris, Mark Gompels, Sue Allan, Adrian Palfreeman, Adam Lewszuk, Stephen Kegg, Victoria Ogunbiyi, Sue Mitchell, Phillip Hay, Christian Kemble, Olanike Okolo, Benjamin Watts, Fabiola Martin, Sarah Russell‐Sharpe, Janet Gravely, Christine Brewer, Sris Allan, Andrew Harte, Debra Brain, Anjum Tariq, Ron Jones, Liz Radford, Sarah Milgate, Jillian Pritchard, Shirley Cumming, Claire Atkinson, Dushyant Mital, Veronica Edgell, Julie Allen, Andy Ustianowski, Cynthia Murphy, Ilise Gunder, Ashini Fox, Howard Gees, Michelle Mieszek, Helen Whitworth, L Anderson, Rajesh Hembrom, Jennifer Teke, Roberta Box, Tom Hatton, Christine LeGegarat, Lee Tomlinson, Ashley Price, Ian McVittie, Victoria Murtha, Laura Shewan, Ade Apoola, Zak Connan, Luke Gregory, Kathleen Holding, Victoria Chester, Trusha Mistry, Catherine Gatford, Valerie Delpech, Roy Trevelion

**Affiliations:** ^1^ MRC Clinical Trials Unit at UCL University College London (UCL) London UK; ^2^ National Institute for Health Research (NIHR) Health Protection Research Unit (HPRU) in Blood‐Borne and Sexually Transmitted Infections UCL in partnership with Public Health England London UK; ^3^ Institute for Global Health UCL London UK; ^4^ St George’s University Hospitals NHS Foundation Trust London UK; ^5^ Imperial College Healthcare NHS Trust London UK; ^6^ King’s College Hospital NHS Foundation Trust London UK; ^7^ University Hospitals Birmingham NHS Foundation Trust Birmingham UK; ^8^ Imperial College London London UK

**Keywords:** AIDS, HIV, mortality, transition, young people

## Abstract

**Objectives:**

To investigate risk of AIDS and mortality after transition from paediatric to adult care in a UK cohort of young people with perinatally acquired HIV.

**Methods:**

Records of people aged ≥ 13 years on 31 December 2015 in the UK paediatric HIV cohort (Collaborative HIV Paediatric Study) were linked to those of adults in the UK Collaborative HIV Cohort (CHIC) cohort. We calculated time from transition to a new AIDS event/death, with follow‐up censored at the last visit or 31 December 2015, whichever was the earliest. Cumulative incidence of and risk factors for AIDS/mortality were assessed using Kaplan–Meier and Cox regression.

**Results:**

At the final paediatric visit, the 474 participants [51% female, 80% black, 60% born outside the UK, median (interquartile range) age at antiretroviral therapy (ART) initiation = 9 (5–13) years] had a median age of 18 (17–19) years and CD4 count of 471 (280–663) cell/μL; 89% were prescribed ART and 60% overall had a viral load ≤ 400 copies/mL. Over median follow‐up in adult care of 3 (2–6) years, 35 (8%) experienced a new AIDS event (*n* = 25) or death (*n* = 14) (incidence = 1.8/100 person‐years). In multivariable analyses, lower CD4 count at the last paediatric visit [adjusted hazard ratio = 0.8 (95% confidence interval: 0.7–1.0)/100 cells/μL increment] and AIDS diagnosis in paediatric care [2.7 (1.4–5.5)] were associated with a new AIDS event/mortality in adult care.

**Conclusions:**

Young people with perinatally acquired HIV transitioning to adult care with markers of disease progression in paediatric care experienced poorer outcomes in adult care. Increased investment in multidisciplinary specialized services is required to support this population at high risk of morbidity and mortality.

## Introduction

With advancements in antiretroviral therapy (ART) and subsequent declines in morbidity and mortality, the life expectancy of adults living with HIV in many settings is nearing that of the general population [1, 2]. Similar declines in AIDS events, hospitalizations and mortality in children living with perinatally acquired HIV (PHIV) have also been reported, in particular from high‐income settings [3, 4, 5]. However, less is known about the impact of lifelong exposure to HIV and ART on mortality as this group reaches adolescence and adulthood, and transitions to adult care [6].

In 2015, mortality associated with HIV was the eighth leading cause of adolescent deaths globally [7], but in many settings, following children as they move through paediatric care and transition to adult care is challenging for several reasons, including disjointed health systems and lack of capacity to conduct longitudinal research. Of the studies that have measured mortality in adult care [8, 9, 10, 11, 12], some are smaller cohorts that may not be representative of the wider population of young people living with PHIV in their respective setting. The UK benefits from a national paediatric cohort, which includes all children diagnosed with HIV and in paediatric care, embedded within the National Health Service, and an ongoing large observational cohort of adults receiving care for HIV [13, 14], We conducted data linkage of these two cohorts, which enabled us to assess the incidence of AIDS events and mortality, as well as identify any risk factors, among young people with HIV who transitioned from paediatric to adult care across clinical settings in the UK.

## Methods

### Study design and participants

In the UK, all children born to women with HIV, and those diagnosed with HIV aged < 16 years are reported to the National Surveillance of HIV in Pregnancy and Childhood (NSHPC). They are subsequently followed through paediatric care in the Collaborative HIV Paediatric Study (CHIPS), although follow‐up ceases when they transfer to adult care [13, 15], The UK Collaborative HIV Cohort (CHIC) Study is an ongoing study collecting clinical data of adults aged ≥ 16 years accessing HIV care from some of the largest HIV clinics in the UK [14], and thus includes any young people followed in CHIPS who have transferred to one of the 25 participating adult clinics. Deaths are reported to the UK CHIC Study by participating clinics and additional mortality reports are obtained through annual linkage to national surveillance reports coordinated by Public Health England (PHE). Both CHIPS and the UK CHIC Study have NHS research ethics approval.

### Statistical methods

Records of young people aged ≥ 13 years by 31 December 2015 who had received paediatric care in the UK and been followed in CHIPS were linked to their respective adult care records in UK CHIC (with data available to 31 December 2015) using deterministic data linkage. A multi‐step linkage process was used which included combinations of the following variables: date of birth, sex, Soundex (a non‐unique code derived from patient’s surname [16]), clinic identifier, clinic name and initials. Validation of linked records was conducted using additional demographic and clinical variables. As only participants with linked records and one or more completed adult care visit at a UK CHIC clinic were included in subsequent analyses, characteristics of this group were compared with young people who had transferred to adult care but who were not identified in the UK CHIC linkage, to assess representativeness. Proportions were compared using the χ^2^ test, and medians using the Mann–Whitney test.

We used a composite endpoint of a new clinical AIDS event or death occurring after the date of the last paediatric visit, rather than a single endpoint of death, due to the reassuringly small number of deaths observed. Demographic and clinical characteristics at the last paediatric visit were compared between those with and without the composite endpoint, and causes of death, clinical and ART status at time of death were also described. Time at risk was calculated from the date of the last paediatric care visit to the date of the first new AIDS event or death, with follow‐up being right‐censored on the last date of adult follow‐up or the administrative censoring date of 31 December 2015 (whichever was earliest) for those who did not experience one of these events. The cumulative incidence per 100 person‐years of a new AIDS event or death in adult care was assessed using the Kaplan–Meier estimator.

Factors associated with a new AIDS event or death were identified using Cox regression. Sex, age at HIV diagnosis in the UK, and calendar year of the last paediatric care visit were included as *a priori* factors in all models, regardless of statistical significance. Age at ART start and calendar year at ART start were not included in the models due to their high correlation and issues of collinearity with age at HIV diagnosis; the variables included were considered more influential on outcomes post‐transition. Other potential risk factors were identified using a stepwise backwards elimination approach with an exit probability of 0.05. Sociodemographic factors considered were ethnicity (black *vs*. white/other) and country of birth (UK *vs*. abroad). Additionally, the following indicators of clinical status at last paediatric care visit were considered: nadir CD4 and CD4 count, viral load > 400 copies/mL in the previous year, AIDS diagnosis in paediatric care, ART status, and the duration between the last paediatric and first adult visit. All analyses were carried out in STATA v.14 (StataCorp, College Station, TX, USA).

## Results

Of 1683 eligible young people in CHIPS, 546 (32%) were linked to records in the UK CHIC Study, of whom 474 were confirmed in CHIPS as having transferred to adult care. Among the remaining 1137 young people with records not linked to the UK CHIC dataset, 479 were documented in CHIPS to have transferred to adult care. Characteristics of both groups are presented in Table 1. Of those with records that were linked to the UK CHIC Study, the vast majority (96.2%) had perinatally acquired HIV, half (50.8%) were female, four‐fifths (80.2%) were black and two‐thirds (60.4%) were born outside of the UK. Median (interquartile range, IQR) calendar year of birth was 1993 (1990–1995), and median age at HIV diagnosis in the UK was 6.0 (2.2–10.7) years. Almost 92% of children started ART in paediatric care, and median age at ART start was 8.8 (4.8–12.5) years.

**Table 1:** Demographic, antiretroviral therapy (ART) and clinical characteristics at baseline and last paediatric clinic visit by linkage status to the UK Collaborative HIV Cohort (CHIC)

	Total (*n* = 953)	Linked to UK CHIC (*n* = 474)	Not linked to UK CHIC (*n* = 479)	*P* value
	*n* (%) or median (interquartile range)			
Demographic and ART baseline				
Perinatally acquired HIV	870 (95.9)	435 (96.2)	435 (95.6)	0.63
Female sex	503 (52.8)	241 (50.8)	262 (54.7)	0.23
Black ethnicity	738 (77.4)	380 (80.2)	358 (74.7)	0.05
Born abroad	558 (61.5)	281 (60.4)	293 (62.3)	0.52
Calendar year of birth	1994 (1991,1996)	1993 (1990–1995)	1995 (1992–1997)	< 0.001
Age at HIV diagnosis (years)	6.6 (2.5–11.0)	6.0 (2.2–10.7)	7.3 (3.0–11.1)	0.05
Started ART in paediatric care	868 (91.1)	434 (91.6)	434 (90.6)	0.61
Combination ART regimen at ART initiation	575 (60.3)	270 (57.0)	305 (63.7)	0.04
Age at ART initiation (years)	8.8 (4.7–12.6)	8.8 (4.8–12.5)	8.9 (4.4–12.8)	0.78
Last paediatric clinic visit				
Age (years)	17.7 (16.8–18.5)	17.7 (16.7–18.5)	17.7 (16.9–18.4)	0.99
Calendar year	2012 (2009–2014)	2011 (2008–2013)	2012 (2009–2015)	< 0.001
Nadir CD4 count (cells/μL)	204 (98–310)	200 (90–293)	215 (110–326)	0.05
CD4 count (cells/μL)	500 (317–683)	471 (280–663)	528 (360–697)	< 0.001
Viral load ≤ 400 copies/mL	603 (64.2)	278 (59.9)	325 (68.4)	0.007
Prior AIDS event (CDC C)	247 (25.9)	131 (27.6)	116 (24.2)	0.01
ART status				
Prescribed ART	852 (89.4)	422 (89.0)	430 (89.8)	0.71
ART‐naïve/ off ART	101 (10.6)	52 (11.0)	49 (10.6)	
Duration in paediatric care (years)	11.2 (6.9–15.1)	12.0 (7.4–15.5)	10.4 (6.4–14.8)	0.01
Duration between last paediatric and first adult visits (months)	Not known	3.2 (1.6–6.2)	Not known	

At the last paediatric care visit, the median age was 17.7 (16.7–18.5) years, and median calendar year was 2011 (2008–2013). Median CD4 count was 471 (280–663) cells/μL, and 59.9% overall (66% on ART) had viral load ≤ 400 copies/mL. At the last paediatric visit, a quarter (27.6%) had a prior AIDS event in paediatric care, for which the median (IQR) age at first AIDS event was 6.9 (2.6–11.5) years. Additionally, at this last paediatric visit 89.0% were prescribed ART, and of the 11% who were not on ART, 76% (39/51) were ART‐naïve. Median (IQR) duration of follow‐up in paediatric care was 12 (7–16) years, and young people had a median gap in care between their last paediatric visit and first adult visit of 3 (2–6) months.

Young people with records linked to the UK CHIC Study were largely similar to young people without linked records; the key differences were that those with linked records were born in earlier calendar years and had their last paediatric clinic visit in earlier calendar years (both *P* < 0.001), and the median CD4 count and proportion virally suppressed at last paediatric visit were lower (*P* < 0.001 and *P* = 0.007, respectively).

**Fig. 1:**
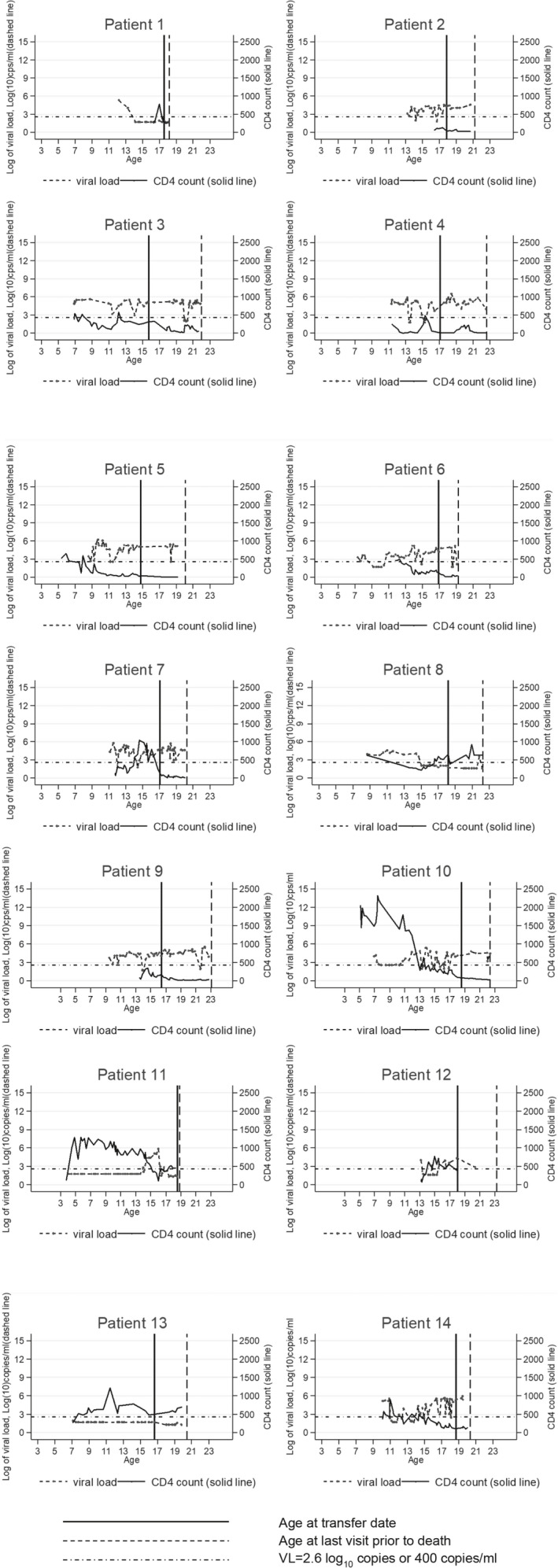
CD4 count, log_10_(viral load) and age at transfer date and death among the 14 young people who died in adult care. Note that similar graphs for seven of the 14 patients who died were published in a previous paper [13]

Of the 474 young people linked to UK CHIC, 14 were excluded as they had no subsequent visits after the first adult visit, so their time at risk could not be estimated. Of the remaining 460, 25 experienced a new AIDS event and 14 died in adult care, with 35 meeting the composite endpoint of AIDS or mortality, during a median (IQR) follow‐up in adult care of 3 (2–6) years. The AIDS events in adult care included candidiasis (*n* = 7, of which six were oesophageal and one unknown), herpes simplex infection (*n* = 1), Kaposi’s sarcoma (*n* = 1), Burkitt’s lymphoma (*n* = 1), pulmonary/disseminated tuberculosis (*n* = 5), *Pneumocystis jiroveci* pneumonia (*n* = 2), pneumonia (*n* = 1), progressive multifocal leukoencephalopathy (*n* = 1), cerebral toxoplasmosis (*n* = 2), HIV wasting syndrome (*n* = 1) and unspecified (*n* = 3). Of the 14 who died, four were diagnosed with a new AIDS event in adult care: one person had their first AIDS diagnosis in adult care at the time of their death, and for the other three the first AIDS event was around 1–4 years prior to death. Causes of death were advanced HIV disease (*n* = 3), HIV wasting (*n* = 1), non‐HIV/AIDS‐related (*n* = 1), suicide (*n* = 1), renal failure (*n* = 1), respiratory disease (*n* = 2), multifocal leukoencephalopathy (*n* = 1) and unspecified (*n* = 4). Nine of the 14 were female, 79% were born abroad, age at death was in the range 18–23 years, and the median (IQR) calendar year of death was 2010 (2009–2013). Deaths occurred a median of 4 (3–5) years following the last paediatric visit. At the last paediatric visit, 12 (86%) of those who died had been prescribed ART, four (29%) had a viral load ≤ 400 copies/mL and the median (IQR) CD4 count was 150 (83–383) cells/μL. By the date of the last adult care visit prior to death (with the date of last visit being the same as the date of death for 11 of the 14 who died), 10 of the 14 were being prescribed ART, and the median (IQR) CD4 count was 35 (15–200) cells/μL. The viral loads of most of the 14 young people who died were uncontrolled before, and continued to increase after, transition, although many had persistently low CD4 counts, often nearing 0 cells/μL, in adult care (Fig. 1).

### Incidence of AIDS and mortality in adult care and associated risk factors

**Fig. 2:**
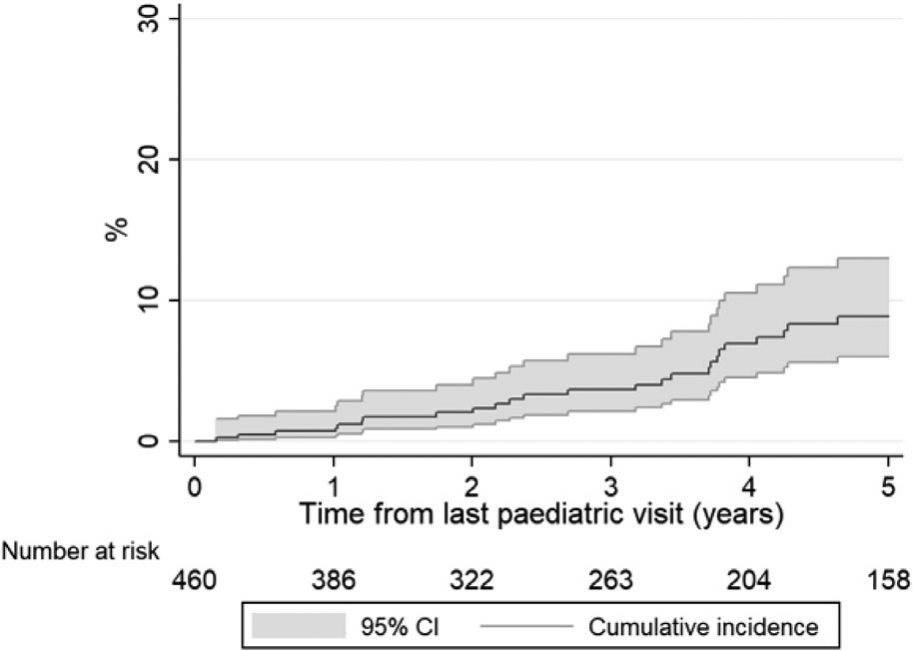
Cumulative incidence of AIDS/mortality in adult care.

Overall, the crude mortality rate was 0.6/100 person‐years [95% confidence interval (CI): 0.4–1.1)] and the rate of new AIDS events/mortality was 1.8 (1.3–2.5)/100 person‐years. The incidence of new AIDS events/mortality increased steadily over the first 5 years of adult care (Fig. 2) and rates were 1% (0–2%), 4% (2–6%) and 9% (6–13%) by 1, 3 and 5 years of adult follow‐up, respectively (Fig. 2).

Table 2 presents rates of, and factors potentially associated with, new AIDS events /mortality in adult care. In univariable analyses, rates were lower in those who transferred to adult care in later calendar years, those born in the UK, and those with a higher nadir CD4 or CD4 count at last paediatric visit. In addition, rates were higher among those with a viral load > 400 copies/mL in the last year of paediatric care and in those with an AIDS diagnosis in paediatric care. In the multivariable model, *a priori* factors (sex, age at HIV diagnosis, calendar year of last paediatric visit) were not associated with the outcome. Of the other potential factors, lower CD4 count at last paediatric care visit [adjusted hazard ratio (aHR) (95% CI) = 0.8 (0.7–1.0)/100 cells/μL increase, *P* = 0.03] and an AIDS diagnosis in paediatric care [aHR = 2.7 (1.4–5.5), *P* = 0.004] remained significant after adjustment for other factors.

**Table 2:** Rates and risk factors of one or more new AIDS event or mortality in adult care

Variable	No. of events	Person‐years (PY)	Rate per 100 PY (95% CI)	Unadjusted hazard ratio (95% CI)	*P‐*value	Adjusted hazard ratio (95% CI)	*P‐*value
*A priori*							
Sex							
Male	13	925	1.4 (0.8–2.4)	1		1	
Female	22	1019	2.2 (1.4–3.3)	1.5 (0.8–3.1)	0.21	1.5 (0.7–3.0)	0.25
Age at HIV diagnosis, per year older	–	–	–	1.0 (1.0–1.1)	0.38	1.0 (1.0–1.1)	0.39
Calendar year of last paediatric visit, per year later	–	–	–	0.9 (0.8–1.0)	0.002	0.9 (0.9–1.0)	0.14
Demographic							
Ethnicity							
Black	31	1510	2.1 (1.4–2.9)	1			
White/other	4	395	1.0 (0.4–2.7)	0.5(0.2–1.4)	0.18		
Country of birth							
Abroad	27	1142	2.4 (1.6–3.4)	1			
UK	8	758	1.1 (0.5–2.1)	0.4 (0.2–1.0)	0.04		
Last paediatric care visit							
CD4 nadir, per 100 cells/μL increase	–	–	–	0.8 (0.6–1.0)	0.07		
CD4 count, per 100 cells/μL increase	–	–	–	0.8 (0.7–0.9)	0.002	0.8 (0.7–1.0)	0.03
Viral load > 400 copies/mL in previous year							
No	8	7520	1.1 (0.5–2.2)	1			
Yes	27	1137	2.4 (1.6–3.6)	2.2 (1.0–4.8)	0.05		
AIDS diagnosis in paediatric care							
No	16	1426	1.1 (0.7–1.8)	1		1	
Yes	19	519	3.7 (2.3–5.7)	3.3 (1.7–6.5)	0.001	2.7 (1.4–5.5)	0.004
ART status							
On ART	30	1658	1.8 (1.3–2.6)	1			
ART‐naïve/off ART	5	287	1.7 (0.7–4.2)	1.1 (0.4–2.8)	0.87		
Gap between last paediatric and first adult visit, per month longer	–	–	–	1.0 (0.98–1.01)	0.72		

ART, antiretroviral therapy; CI, confidence interval.

## Discussion

Our study, using linked data from the CHIPS and UK CHIC Study cohorts, is one of the largest multi‐centre studies of young people with perinatal HIV transitioning from paediatric to adult care. We reported a high incidence of new AIDS events/mortality in adult care for this population. Choice of this composite endpoint enabled us to investigate associations with socio‐demographic characteristics of these young people as well as their characteristics at their last paediatric care visits. We showed that young people at greatest risk of experiencing a new AIDS event or death following transfer to adult care were those who had an AIDS diagnosis in paediatric care, or who had a lower CD4 count at their last paediatric care visit. Our findings highlight the clinical complexities of this cohort, with nearly 1 in 10 people experiencing an AIDS‐defining event or dying by 5 years’ follow‐up in adult care.

Overall, 35 people in our analysis experienced a new AIDS event or died after transitioning to adult care. The mortality rate in our study, of 0.6/100 person‐years, is more than 12 times higher than the mortality rate in the general population of people aged < 25 years in the UK (0.03–0.05/100 person‐years) [17], highlighting the ongoing vulnerability of our cohort despite widespread ART access. Our rate of adverse outcomes is lower than a study of 735 young people with PHIV in New York who transitioned to adult care at a median age of 22 years [11]. In that study, the mortality rate was 5.6/100 person‐years in the first year after transition to adult care, with most deaths in the first 6 months of adult care. By contrast, most deaths in our study occurred more than 3 years after the last paediatric care visit. However, there were differences between the two studies in terms of age at transition to adult care and healthcare systems. The New York cohort transitioned at a median age of 22 years, so were older than our cohort at the time of transition to adult care. Furthermore, the New York patients who transitioned to adult care had poorer health status than those who remained in paediatric care, with paediatric care continuing to be available up to age 25 years in some settings and populations in the USA [18]. Our analysis updates a previous mortality study among young people with PHIV attending 14 clinics in England, which reported eight deaths in adult care, some of which may also be included in our findings [10], The previous study found a mortality rate in adult care of 0.5 and 0.9 for those aged 16–20 years and ≥ 21 years, respectively, similar to what we report here.

In our study, the two significant predictors of AIDS and mortality in the multivariable analysis were lower CD4 count at last paediatric care visit and having had an AIDS diagnosis in paediatric care (at median age 7 years), which themselves indicate suboptimal health outcomes in paediatric care. Lower CD4 count at last paediatric care visit is an indicator of young people who struggle to manage their HIV infection, and these young people may benefit most from more intense interventions prior to and continuing into adult care. Longer‐term follow‐up is needed to evaluate mortality trends among adults with PHIV as they reach older ages. In the previous study in England [9], it was noted that all young people who died had potentially suppressive ART regimens available to them, and many had documented adherence problems in paediatric care, although in this analysis resistance data were not available. Other research suggests that HIV may contribute to neurological changes which themselves may affect outcomes such as adherence [19]. Additionally, as brain maturation completes at around 25 years of age, it may be the case that adherence improves and mortality starts to decline in young people with PHIV who survive to their mid‐20s, establish regular ART treatment habits and achieve full viral suppression [20, 21, 22].

Our study had several limitations. First, the 21 clinics that participate in the UK CHIC study include several specialized, tertiary‐care HIV clinics located in London and the south‐east of the UK, potentially limiting the generalizability of our findings to non‐tertiary‐care settings and other UK regions. We tried to address this potential bias by comparing characteristics of young people who had transitioned to adult care by whether they had been linked to the UK CHIC Study or not, and CD4 counts and viral load at transfer were, on average, poorer among those going to UK CHIC clinics. Consequently, our study may have some selection bias in favour of young people with poorer health who may have been more likely to be referred to tertiary clinics with better adolescent care provision, which may therefore result in overestimates of rates of AIDS events and mortality. Our study required young people to have at least one adult visit, and thus the design prevented us from measuring mortality in young people who transitioned to non‐UK CHIC clinics, or those who may have died before they had an adult care visit, or compare mortality with that immediately pre‐transition. We were also limited to routine clinical record data in our exploration of risk factors for AIDS and mortality. However, as suggested by Fish *et al*. [10], AIDS and mortality may be driven by a multitude of other factors, such as mental health and ART adherence. Additionally, our analysis was limited to factors at last paediatric care visit which were associated with AIDS/mortality, while future analyses might take a time‐updated approach to ART, viral load and CD4 measures. Further, our ART measure may have been subject to misclassification if young people being prescribed ART and therefore categorized as ‘on ART’ in our analysis were, in reality, not taking it. Lastly, given the small number of events, our multivariable model may be overfitted (five covariates in the final model) which could reduce the accuracy of the exposure estimates. However, the major strengths of our study were the relatively large sample size and the multiple clinics included.

In conclusion, our study found that young people with PHIV who transitioned to adult care with poorer immunological markers and a history of more advanced disease progression were at increased risk of AIDS events and death in adult care. A majority of young people with perinatal HIV who died experienced consistently poor immunological and virological control during their paediatric and adult follow‐up. It does not appear, therefore, to be transition *per se* that results in poorer ART adherence, but instead a continued pattern of suboptimal adherence established in early adolescence while in paediatric care. Investment in appropriate and relevant multidisciplinary support services in paediatric and adult HIV care is essential to minimize this population’s high risk of morbidity and mortality, not to mention the potential for onward HIV transmission.
